# Death of Mrs. Wm. F. Holt

**Published:** 1884-07

**Authors:** 


### DEATH OF MRS. WM. F. HOLT.

It is with profound sorrow that we announce the death of this estimable lady—wife of Dr. Wm. F. Holt, of Macon—which occurred at her home a few weeks since. We extend to Dr. Holt and family our heartfelt sympathies in this their sad bereavement.

Below we publish resolutions of sympathy adopted by the Macon Medical Society:

At a meeting of the Macon Medical Society, held Tuesday evening, June 3d, a committee was appointed to draft resolutions of sympathy for Dr. Wm. F. Holt and family in their recent bereavement. The following resolutions were presented and unanimously adopted:

"*Resolved* (1), That the Macon Medical Society has heard with profound regret and sorrow of the death of the accomplished wife of our friend and fellow laborer, Dr. Wm. F. Holt.

"*Resolved* (2), That we tender to Dr. Holt and his family our sincerest sympathies and condolence, in this, their sad bereavement and irreparable loss.

"*Resolved* (3), That a page upon the minutes of this society be set apart for these resolutions and they be spread thereon.

"*Resolved* (4), That a copy of these resolutions be furnished to Dr. Holt and family, and also to the *Telegraph and Messenger* with the request that they be published.

JOHN E. BLACKSHEAR.
K. P. MOORE,
H. J. WILLIAMS,
Committee.
C. H. HALL, President.

HOWARD J. WILLIAMS, Secretary.

